# Cockade structures as a paleo-earthquake proxy in upper crustal hydrothermal systems

**DOI:** 10.1038/s41598-019-45488-2

**Published:** 2019-06-25

**Authors:** Alfons Berger, Marco Herwegh

**Affiliations:** Institute of Geological Science University Bern, Baltzerstr. 1+3, 3012 Bern, Switzerland

**Keywords:** Tectonics, Solid Earth sciences, Structural geology

## Abstract

Cockades are clasts completely surrounded by spheroidal hydrothermal overgrowth rims. They are observed inside hydrothermal fault breccias and can provide insights into fault dynamics. The formation of cockades with spheroidal hydrothermal overgrowth rims is related to fast fracturing and dilation, and requires primary clasts to be suspended in a fluid. The rim growth is driven by drops in fluid pressure and related oversaturation. We use descriptions of cockades, their rims and cements in a fault breccia. Geometrical data are combined with mechano-chemical calculations to gain insights into seismic processes and estimate seismic magnitudes. Fast rates for formation of cockade cores and first rim growth are interpreted to be the result of an earthquake’s main shock. Younger growth rims represent subsequent aftershocks, while cemented cockades record interseismic periods. We propose that by considering growth rates of hydrothermal precipitates and cements, paleo-earthquake cycles can be unraveled and a link between geophysics and fault structures can be established.

## Introduction

Seismic activity is one of the major natural hazards affecting communities across the globe and considerable scientific effort is directed towards understanding earthquake phenomena to mitigate potential disasters. Geophysical surveys by seismometers and GPS stations are state of the art techniques used to monitor ambient seismicity at a variety of scales^1^. These techniques can be used to depict the seismogenic zone in different rocks, which, in the continental crust and non-subduction zone plate boundaries, can reach down to 15–20 km. Despite providing accurate information on the location, depth, energy release and timing of earthquakes, the aforementioned geophysical techniques cannot currently provide high enough spatial resolution at the millimeter to meter scale to study the full range of geological processes related to the seismic cycle. The spatial scales at which the earthquake producing processes mentioned above act, are on the meter to submicron scale and the time scales vary significantly from seconds up to ten thousands of years^1^. Therefore, to gain information about these processes active during the seismic cycle, both rock deformation experiments and the study of ancient seismic activity recorded in exhumed natural rocks are needed^2,3^. This contribution focuses on the rock record of seismicity in exhumed natural fault rocks of the Grimsel Breccia Fault (Central Switzerland).

Although (paleo-)seismic activity may be recorded by clear near-surface evidence (e.g. active fault scarps, fault trenches, mole tracks, surface fissures, offsets), proving its existence is challenging in the case of exhumed fault rocks. To date, potential indicators for seismic events in exhumed natural rock records are: (1) pseudotachylites generated by earthquake-induced shear heating in the relatively dry deep crust and upper mantle^2,4–6^; (2) fluidization textures in soils or fault rocks^7,8^; (3) evidence for frictional devolatilization such as CO_2_ release in carbonates or H_2_O release in hydrous silicates^9–12^; (4) the frictional-heating induced formation of layers of amorphous material or nanocrystallites^9,11,13^; (5) maturation of organic material by a combination of strain and shear heating^14,15^; as well as (6) indirect evidences that is interpreted to be related to earthquakes^5,16^. Unfortunately, an unambiguous proof of the seismic origin of such features is impossible since subseismic rates can also result in similar structures^17^. To provide conclusive evidence for seismic activity, clear evidence of processes occurring at rates of 0.1–1 ms^−1^ ^18^ would be of great help, although seismic slip rates may even go down to 10^−4^ ms^−1^ ^5^. The current study deals with such high-speed processes occurring at depth in basement rocks during fluidization. In addition to brecciation and cataclasis, which may be related to earthquakes or not^19,20^, different fluidization processes have been proposed to take place during seismic activity^7,8,21,22^. The term fluidization has been used for solid-dominated material containing pressurized fluids. The resulting fluidized material can deform at variable rates, which can span orders of magnitudes from mm/s to several m/s. In the following, the term fluidization is applied for rock/mineral fragments being in a fluid-dominated suspension^7^. This type of fluidization depends on porosity, fluid viscosity, fluid composition and potential pressure drops^7^ (Appendix 1). Importantly, this allows a direct and useful link to be made between detailed field descriptions of rocks and the properties obtained by geophysical surveys using the propagation of p-, s- and surface-waves.

In this study, we investigate an exhumed hydrothermal system in the Aar Massif (Central Alps, Switzerland)^23–27^ to unravel the link between seismic events and cockades found in a fossil fault breccia. The understanding of this rock record requires data on: (1) geometry (size of the fault structure), (2) stress/pressure evolution; and (3) fluids (composition, amounts, solubility of elements as well as flow velocities). The geometry and microstructures can be quantitatively analyzed, whereas stress/pressure evolutions have to be calculated from physical considerations. We show how such hydrothermal field structures can be used as earthquake proxies to characterise earthquake behavior. The associated data and calculations are provided in the appendix.

## Observations and Implications

### Field relations and geological context

**Figure 1:**
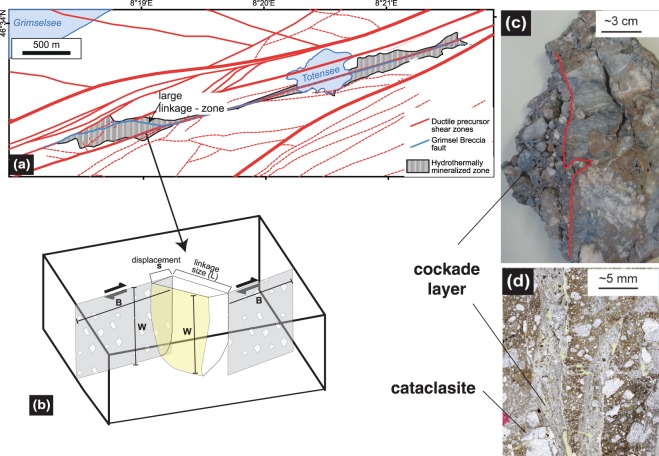
Situation of development of hydrothermal cockade textures. (**a**) Geometry of a strike slip linkage zone (modified from^24^). (**b**) The size of the linkage zone is defined by the displacement (s), the size (B), and the vertical width (W). The active fault is defined by the complete length of the fault and the width (W); (**c,d**) natural examples of preserved cockades; (**c**) hand-sample, (**d**) thin section photomicrograph.

**Figure 2:**
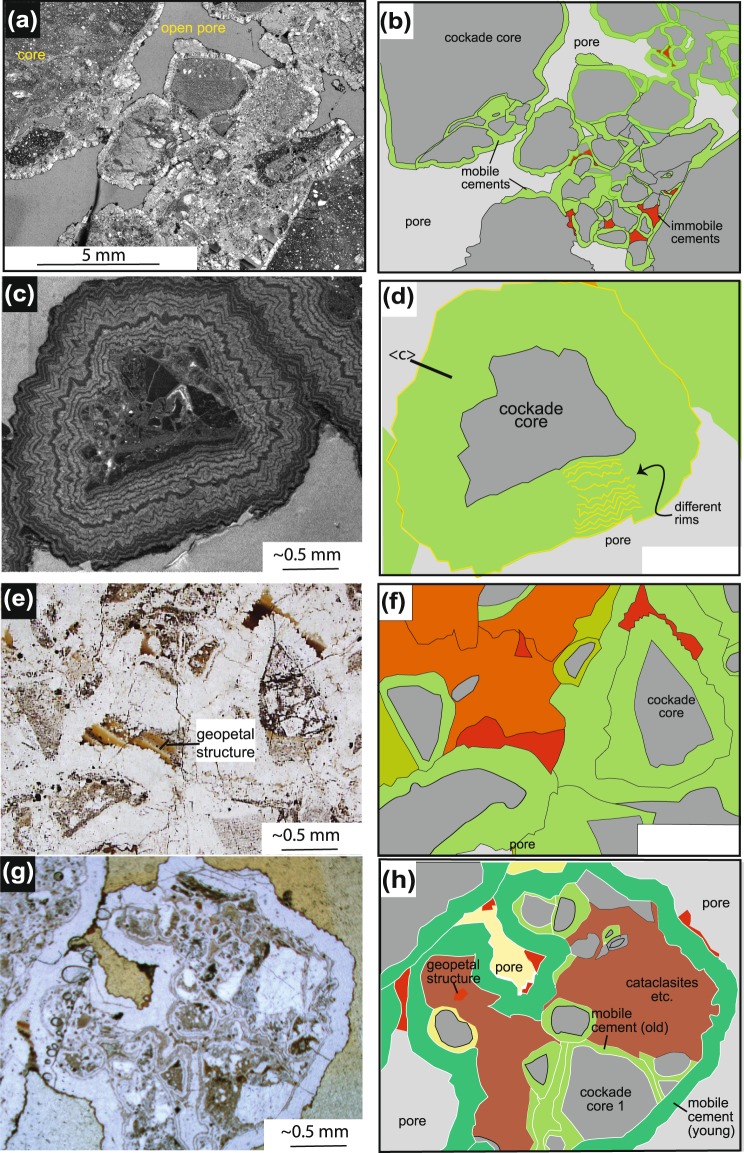
Textures of cockades of the Grimsel breccia fault. (a) Overview of such a breccia showing the high porosities (photomicrograph of a thin section in plane polarized light); (**b**) drawing of (**a**). (**c**) Catholuminesence image of one cockade particle, (**d**) drawing of (**c**) indicating the different growth rims and the growth direction of the quartz with the quartz <c> axes; (**e**) cemented area of the cockade breccia (plane light); (**f**) hand drawing of (**e**) showing the cockades and the different types of cements. One group of cements are cherts with geopetal textures; the second group of immobile cements are large adularia crystals filling pore space or fine particles and chalcedony filling space (**g**) optical photomicrograph (plane light) of cockade textures inside cockades, (**h**) drawing of (**g**), the bright green colors are cockade rims of an older generation, which are partly cut by the next rupture event.

The Grimsel Breccia Fault (GBF) is situated in the Southwestern-Aare granite, and originates from strands of the former crustal-scale ductile Grimselpass shear zone^23–27^ (Fig. 1a). This shear zone initially evolved during Alpine deformation at depths of ~20 km some 22–20 Ma ago^26^. Resulting banded mylonites are generally characterized by fine-grained (<100 µm) polymineralic layers dominated by two feldspars, quartz, mica and epidote, intercalated with dynamically recrystallized quartz bands. Viscous granular flow (diffusion creep) is the dominant deformation mechanism in the polymineralic bands, although the occurrence of injection structures recording temperatures >400 °C suggests periods of seismic activity already occurred at these depths in the Grimsel shear zone^25^. In close vicinity, gneissic textures with similar mineralogy, but larger grain sizes (mm - cm), are observed. Grain-size dependent viscous deformation led to strain localization along different shear zone strands^24,25,28^. The shear zones originated as reverse faults with movement of south-block up kinematics, which changed to an overall dextral strike-slip framework some 14–12 Ma^25,26^. With progressive Alpine orogeny, the shear zone was exhumed to shallower crustal levels and at 7–5 Ma a transition from dominantly ductile to brittle deformation occurred^26,29,30^. Consequently, cataclastic deformation overprinted the aforementioned mylonites and gneisses. With progressive exhumation, increasing volumetric strain and hydrothermal activity occurred, forming fault breccias and cockades, during ongoing dextral strike-slip faulting that lead to the Grimsel Breccia Fault (Fig. 1a). This brittle fault system consists of subvertical WSW-ENE striking master fault segments of several kilometers (>4.6 km)^24^ length (Fig. 1a). The array of master faults is connected by linkage zones (Fig. 1a,b), which occur at several scales ranging from the km- down to the mm-scale. The associated fault rocks of the brittle network show characteristic strain-dependent transitions from tectonic breccias to ultracataclasites, where multiple cycles of embrittlement, cementation and refracturing can be observed (Figs 1c,d and 2). The largest of the linkage zones have been mapped^24^ and the smallest are inferred from hand-samples and thin-sections (Fig. 1c,d). In rare cases, the total accumulated horizontal displacements along individual large-scale brittle strands have been estimated to be on the order of 25–45 m^24^. Note, however, that the individual displacement increments were in the range of millimeters to centimeters. In the case of cockade breccias, the scale of displacement can be inferred from the size of the apertures, which must have opened at seismic rates as will be demonstrated below.

During the entire fault network’s activity, fluids were present and substantially affected deformation. One consequence of this fluid circulation is that a major hydrothermal cell has been active since at least 3.4 Ma^23^ resulting in today’s highest Alpine hot spring (elevation of 1908 m) with discharge temperatures of 28 °C^23,31,32^. The aforementioned fault linkage structures facilitate the ascent of the hot hydrothermal waters^24^. Fluids rise through the linkage structures, which are subvertical tube-like systems, due to their enhanced permeability^24^. It is within these fractured parts of the fault linkages that the cockades being suggested as earthquake proxies can be found.

### Microstructural observations

**Figure 3:**
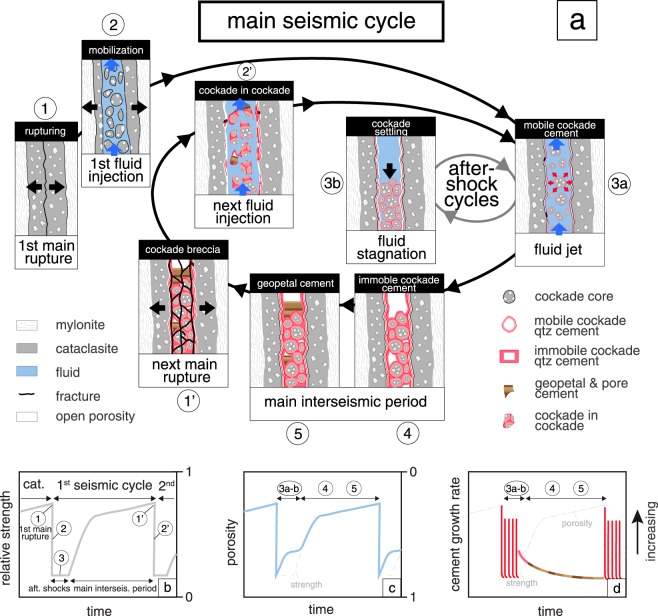
(**a**) Schematic sketch showing a main seismic cycle of cockade breccias starting from initial fracturing (1) of a cataclasite during fluid injection/particle mobilization (2) and simultaneous growth of mobile cockade cements owing to an isoenthalpic pressure drop (3). Secondary aftershock cycles result in multiple growth events of mobile cockade cements (3a-3b). In periods of reduced fluid velocity the cockades settle and immobile cockade cements (4) followed by geopetal cements (5) precipitate before the next main rupture induces a new seismic cycle (1′). (**b**–**d**) Link between inferred changes in relative strength of the fractured tectonite (**b**), associated porosity changes in the created fracture (**c**) as well as (**d**) the sequence of different cement types and associated relative growth rates.

Our earthquake proxy is based on cockade-breccias, which occur in linkage zones of the GBF (Fig. 2a). The term cockade-breccia is used to refer to hydrothermal fault infills of clasts completely enclosed by concentric bands of hydrothermal minerals^7,33,34^. The transition from the host granite to fully consolidated cataclasites and then to cockade layers with their open porosity occurs entirely in the GBF (Figs 1, 2d,e and 3). Individual cockade clasts are derived from fragments of cataclasite (Figs 2a,c and 3). These cores are then surrounded in 3D by monomineralic, but polycrystalline quartz-growth rims (Fig. 2a–d, Appendix 2, Fig. A2). Therefore we can infer that the cockade cores must represent a long history: an earlier high strain deformation, its cessation, cementation and subsequent fragmentation (Fig. 3). The documented complete surrounding of the cockades by a 3D rim of precipitated quartz implies mobilization and free suspension of the cockades in an ascending fluid jet that is oversaturated in SiO_2_ (Fig. 3). The measured growth rims have widths of 0.2–4 mm (Fig. A1) and consist of several growth layers (containing a low + high cathodoluminescence (CL) intensity rim), each showing a width between 20–30 µm (Fig. 2c,d). Electron microprobe analyses reveal that the individual growth layers are visible due to changing Al/Si ratios within the quartz grains as is most clearly observed in CL imaging (Fig. 2c). Within rims, the individual quartz grains are rod-shaped with their <c> axes being radially arranged around the cockade core^7,33^. They proceed as single grains from the inner to the outermost layer. Hence it can be interpreted that each new growth layer developed without nucleation, but by subsequent growth of existing quartz grains resulting in a new 3D spheroidal layer that completely encloses the former one. This means that at least during formation of a growth layer, the cockade was in suspension and attached neither to other cockades nor the wall rock (Fig. 3). We therefore attribute the growth rims to a mobile cockade cement stage (Figs 2 and 3).

There exists a second, distinct, type of quartz cement, which overgrows entire cockade aggregates (Fig. 2g,h). This implies an end to suspension and the settling of the cockades to the bottom of the fracture. Here, the cockades remained motionless long enough to be covered by the second cement type, which we refer to as immobile cockade cementation (Figs 2 and 3). We interpret this microstructural relationship associated with settling to indicate a reduction in the fluid velocity. By this second cementation stage, the cockade aggregates gain cohesion. But the volume occupied by this cockade-particle-supported framework still suggests occurrence of an interconnected porosity (Figs 2 and 3). In rare cases large, idiomorphically grown adularia crystals can be found in this porosity but more generally these pore spaces become successively filled by chalcedony/quartz and fines that form a layered sedimentation structure typically referred to as a geopetal fabric (Fig. 2e). Therefore, we consider this to be another distinct cement and call this third type of cementation *geopetal and pore cements* (Figs 2e and 3). The sedimentary layering and the small grain sizes (<2 µm) of particles within these layers indicate very low flow velocities or even a stagnant fluid during the formation period of geopetal and pore cements. Fluid inclusion studies by^23^ demonstrate quartz precipitation under low saline conditions at temperatures between 160°–100 °C without boiling.

### Cataclasis versus cockade formation: Loss and gain in fault rock strength

The observations that (i) cockade structures are embedded in cataclastic host rocks, (ii) cockade cores often consist of cataclasite fragments and (iii) deformed cockade structures occur all suggest a close co-genetic evolution, which allows some inferences to be made on the change in mechanical state within these fault rocks. Although cataclasites are defined as cohesive fault rocks once exposed at the Earth’s surface, it is not clear whether they were also cohesive during their deformation at depth, or rather deformed by frictional granular flow processes in a non- or weakly cohesive aggregate state. Based on our samples we can clearly state that at the time of cockade formation the cataclasites must be impermeable and highly cohesive, allowing for hydrofracturing of this fault rock as well as the dragging of cataclasite clasts in the fluid jet (Fig. 3). We therefore speculate that the deformation rate in the cataclasites was not constant. Here stages of frictional granular flow with somewhat enhanced velocity (velocity weakening-strengthening cycles e.g.^35^) and probably reduced cohesive strength alternate with subsequent stages of quiescence when cementation/healing of intergranular pore space resulted in both porosity loss and strengthening^19,36–39^. Owing to the latter two aspects, lithostatic pore fluid pressures can start to build up providing the framework for subsequent hydrofracturing and associated cockade formation, again reducing the cataclasite’s strength.

The cockades themselves also represent a long history closely associated with changes in strength of individual cockades, cemented cockade aggregates or the finally cemented cockade breccia (Fig. 3). Firstly cockade cores are formed during a rapid brittle event resulting in the aforementioned disintegration of the cataclasite. With instantaneous dilatant deformation, the fragments are then suspended in a jet of hydrothermal fluid that allows a spheroidal quartz rim to grow (see below). With time and reducing fluid velocity, the cockade spheres settle and a second cement is precipitated, resulting in agglutination of several cockades. They can either by brought back into suspension by a new fluid injection or undergo final consolidation to a cockade tectonite, by formation of geopetal and pore cements in open pore space (Fig. 3). From a mechanical point of view, it is this last cementation stage that yields a substantial strength gain for both cockade tectonites and neighboring fractured cataclasites (Fig. 3). The observations that the same samples that show new cockade cores also contain fragments of (i) cockades (Fig. 2g), (ii) immobile cements and (iii) geopetal and pore cements (Fig. 2g) clearly emphasizes the repetitive nature of this sequence (Fig. 3).

## Chemo-thermo-mechanical Implications

The cockade-breccia described above is hosted within a dilatant linkage zone of the GBF strike-slip fault system where vertical fluid flow produces the resulting hydrothermal system^23,24^. Necessarily, associated microstructures and the large-scale fault dynamics of the host strike-slip fault system must be closely related to each other. Here we explore this relationship by linking quantification of the large-scale geometry and the microstructure to a series of chemo-thermo-mechanical considerations to obtain important information on the paleo-seismic record of the host fault.

### Record of seismic activity

The formation of the 3D mobile cockade cements demands (i) the generation of high porosity, (ii) cockade cores in suspension (fluid velocity) and (iii) an oversaturation in SiO_2_. The first and the second conditions require the opening of a fracture and fast fluid flow (a fluid jet) capable of mobilizing rock fragments (Fig. 3). All of these conditions are caused by, and can be linked to, seismic rupture. Firstly, the size of the opened fracture in the linkage zone depends on the displacement of the fault (Fig. 1a), which in turn directly relates to the energy release of the related earthquake (Appendix 1). Secondly, the fast velocity of the injecting fluid requires an initial pressurization, which would drop down after the opening of a fracture (Figs 1 and 3; Appendix 1)^24^. This implies a fast energy release indicating a seismic process. In turn, the SiO_2_ oversaturation, which gives rise to the mobile cockade cements, is controlled by the porosity, the velocity of the fluid and the SiO_2_ solubility. Together, these properties can be explored to gain insight into earthquake activity on the fault plane.

The fluid velocity mainly depends on the porosity, the fragment size and fluid viscosity (Appendix 3). Moreover, the porosity and fragment size are highly dynamic, because of the movement of cockades in the fluid jet as well as the growth of cements. For example, the initial porosity (fluid filled space) can be considered as a function of the volume of the fracture (Fig. 1) as well as the number and size of the fragments in the fluid (Fig. 1). With progressive cement growth the size of particles will change, leading to changes in the porosity, which ultimately will all change the fluid velocity (Figs 1 and 3). However, the cement growth depends on the SiO_2_ solubility, which in turn depends on the thermodynamic behavior in the fluid at the time of pressure drop. This behavior is isoenthalpic, meaning that the fluid undergoes cooling at a given pressure drop (Joule-Thomson effect). Both pressure and temperature changes decrease the SiO_2_ solubility, resulting in the immediate precipitation of the mobile cockade cements. These relationships between the different parameters can be illustrated for our selected example (Figs 1–3). In the case of an initial porosity fraction of 0.2 (20 vol%) and an average cockade core diameter of 2.5 mm (see Fig. A1), the fluid/cement ratio is ~5.38. For an isoenthalpic pressure drop (from 160 down to 50 MPa = 110 MPa pressure drop, Appendix 3, Table A3) at a temperature of 150 °C, the SiO_2_ solubility change (ΔC_s_) is 0.000945 mol/kg water. In addition, 1.76 * 10^−5^ mol of quartz is necessary to precipitate the volume (0.39 mm^3^) of one representative mobile cockade cement rim (Fig. A3). These values can now be used to consider the dimensions of the fluid-system in the case of a simplified system consisting of one growth rim around a single cockade core. Taking the aforementioned ΔC_s_, a fluid volume of 18’624 mm^3^ is required to supply enough SiO_2_ for this precipitation. Precluding the seismic rate of rupturing, one can estimate the length of the fracture necessary to carry this fluid amount (see Appendix 3). For this step, the obtained fluid volume needs to be transferred into a 3D framework (l * l * h, Appendix 3). As an example, for a cockade of a diameter (l) of 2.5 mm and the above-mentioned rims, a value of the minimum length (h) of this hypothetical cockade-fluid system will be around 4–8 m (Fig. A4, l is given by the size of the cockade). This value of course needs to be extended, since the microstructures show that our systems contain a large number of cockades with precipitation rims. Therefore a larger fluid volume is required, further expanding the length of the fracture. As already stated by^7^ the fluid/cockade ratio during the suspension stage is dynamic. For our calculation this ratio is crucial, since it defines the expansion of the calculated fracture length as a function of increasing cockade content of the suspension. Therefore, we correct the calculated fracture length for different assumed porosities (equation A16). At this step, we can only assume different porosities for the suspension stage: For different porosities the fracture lengths (W’_min_ = 2*h) decrease from 14.24 m (at porosity: 0.2), to 6.98 m (at porosity: 0.4) to 3.76 m (at porosity: 0.8; Figs. A4 and A5).

Note that these additional cockades also show growth rims and need to be supplied by enough SiO_2_ in the representative fluid volume. Therefore, this volume needs to be expanded by the number of cockades to guarantee sufficient SiO_2_ supply at a given solubility (equation A18). After all these increments, we obtained a fluid column with an attributed height, capable of growing the SiO_2_ rims for all cockades observed in this vertical structure. Assuming an initial porosity directly after the earthquake rupture of 0.8 and using the above measured cockade dimensions, the resulting fracture length (W”) would be 1714 m (=2*h” with h” = 857 m; see Table A3).

Having now established the dimensions of the hydrothermal system active during a single rupture event, constraints on its time frame are required. The Ergun-equation^40^ provides estimates on the velocity of the cockades being suspended in the fluid^7^, which given the aforementioned constraints ranges from 0.07 to 0.11 ms^−1^ for cockade diameters of 1 and 2.5 mm size, respectively. This flow rate, in combination with the estimated length scale, delivers the minimum time available for a mobile cockade rim to grow. In the case of our estimates, the time interval ranges from 1.2 to 0.3 minutes (Table A3). The fluid velocity and rates of rim growth are exceedingly fast and we therefore suggest they require a rupturing at seismic rates and associated injections of fluids into the newly created fracture space.

### Evidences for seismic cycles

The fact that multiple growth rims occur points towards multiple pressure drops. For each rim, the quartz precipitation processes are identical to those mentioned above. Since the rim widths are similar, the changes in pressure drops must be similar as well. Hence, each pressure drop represents a new seismic rupture, where free 3D growth of the cockades in suspension is possible^7,39^.

For the interseismic periods, the cockades can either remain in suspension, which would require a certain sustained fluid flow velocity^41^, or the velocity decreases such that the cockades settle to the base of the fracture. In any case, no mobile cementation occurs during this interseismic period since the cockades are neither attached to the base nor to each other. Therefore relatively short interseismic time intervals^42^ must be assumed and the ruptures have to be related to aftershocks rather than to a new major shock.

This is in contrast to immobile cockade cements. Here overgrowth of several cockades by immobile cockade cements (Figs 2 and 3) indicates a reduction of fluid velocity imposing the settling of cockades. In this way, cockade aggregates gain cohesion and may even attach to the host rock (Figs 2 and 3). Remaining pore space reduces with progressive cementation. A further decrease in fluid flow velocity, i.e. stagnant or a low velocity fluid induces a settling of fines in sediment traps (geopetal structures, Fig. 2e) as well as the precipitation of pore cements (adularia, chalcedony, celadonites, Fig. 2e–h). Given the low flow velocities of the fluid, major pressure drops must be excluded as a driving process for precipitation of the geopetal cements. Minimum estimates on the precipitation time required can be made by using the thickness of geopetal cements, the saturation of the fluid at given temperature conditions and the amount of fluid required, the latter being calculated by the flow velocities during the geopetal cementation (Appendix A4). Based on these premises a precipitation time of 2880 years at a length scale of W” (=1714 m @ initial porosity 0.8) can be estimated, which clearly indicates a major interseismic period. We therefore interpret that this cementation stage represents the end of a major seismic cycle (Figs 2 and 3).

In the case of multiple major seismic ruptures, a refracturing of the cemented cockade texture is necessary. In fact, this stage is documented by fractured and remobilized cockade textures (including geopetal and pore cements) being themselves enclosed by a new generation of mobile cockade cements (Figs 2g,h and 3). The larger dimensions of this second cockade generation compared to the first one suggest a flow velocity higher than observed in previous events (Fig. 2h). In the case of too low fluid flow velocities, no mobilization of the former existing cockade aggregate occurs. At very low fluid velocities a sequence of immobile cockade cements and geopetal/pore cements develops (Figs 2e and A1e).

## Conclusions

Cockade textures with their cores and different cementation stages provide a proxy to unravel paleo-seismic activity along large-scale faults^7^. The proxy requires information from multiple scales: quantified field relations from meter to hundreds of meters scale deliver information on the style of faulting, the kinematics and the major geometric parameters (e.g. length, width, crosscutting relations); while microstructural relationships between cockade cores and cements combined with quantifications of core sizes, cement volumes and their chemistry are used to parameterize seismic events. The first momentum forming a new cockade structure is related to the main shock. Subsequently, the number of overgrowth events represents the number of ruptures (aftershocks), while the geopetal cements give information on the duration of tectonic quiescence, i.e. the interseismic period (equation A25). Note that the presented approach only delivers relative age relationships, which can be calibrated towards absolute ages by applying recently developed radiometric dating methods using radiogenic contents of specific cement minerals such as adularia^23^, calcite^43–45^, or other minerals^39,46^.

The seismic formation of cockade breccias and their different cementation stages within fault zones require the creation of substantial pore space and the presence of considerable amounts of fluid that is injected instantaneously. These facts, in combination with the overprinted cataclastic cores, suggest that this type of cockade formation is related to seismic activity in the upper crust. Given the worldwide abundance of fault-bound hydrothermal systems, fluid-assisted seismic activities have been inferred. With our new tool, time constraints not only on the rupturing but also on the healing/cementation intervals between seismic events can be obtained. Linking quantitative studies on cockades/cataclasite systems with rock deformation experiments can provide important information on (i) the effective chemo-mechanical feedback processes, (ii) evolution of pore fluid pressures and (iii) changes in fault rock strengths. It is these three fundamental parameters that mainly affect the loading of the fault rocks and define whether aseismic creep can persist or tectonic stresses will build up preparing the fault for the next rupture.

In this light, more information may also be extracted from geometrical data from cockade tectonites. Particularly the elevated seismic activity in the vicinity of hot springs might be related to cockade formation. In the case of the study area, the largest reported cockade structures yield maximum paleo-earthquake magnitudes of 4–5 (W”: 1714 m, L: 4600 m, s: 0.1 m, see Table A3 for details). Many smaller cockade examples exist, which would require smaller but more frequent magnitudes. These values are identical to those recently found in active hydrothermal systems of the Valais region^47^. Last but not least, domains of fresh cockades with their grain-supported framework, enhanced porosity and limited cementation by immobile and geopetal cements should sustain a high permeability. Therefore, understanding the dynamics of cockade breccias might be of great interest for the exploration of geothermal energy in active hydrothermal systems.

## Methods

### Image analysis

Microstructural investigations were performed by light- and scanning electron-microscopy (SEM ZEISS EVO50) using variable pressure conditions and different detectors (VPSE: variable pressure electron detector; CL: cathodoluminesence detector; BSE: backscatter electron detector). These images allowed the separation of cockade cores, mobile and immobile cockade cements, geopetal and pore cements as well as still-open porosity by manual line drawing. Thereafter, each particle was analyzed for size, area, perimeter and length of major and minor axis by the software ImageJ 1.46^48^. The resulting area of each particle was transferred into an equivalent diameter^49^. The particle size and other geometrical parameters were then calculated (Appendix 2).

In the interseismic cements the average particle size was estimated by SEM images inside the geopetal cements. The thickness of each infilling was measured in thin section with an optical microscope equipped with a calibrated length measuring tool.

### Solubility calculations

The software “LONER” (www.uni-leoben.at) was used to calculate the differences in solubility values (Δc_S_) for SiO_2_ in an aqueous fluid at changing P and T conditions (Fig. A3). Solubilities were calculated using the equation of state from^50^. For the shock-induced pressure drops we assume isoenthalpic fluid flow and use the Joule-Thomsen factor to calculate the related temperature changes. The relevant Joule-Thomsen factors are borrowed from http://webbook.nist.gov/. The link between concentration changes and measured masses of the immobile cockade cements is given by the volume of the corresponding cockade layer.

## Supplementary information

Supplementary information 1

Supplementary information 2

Supplementary information 3

Supplementary information 4

## References

[1] Fukuyama, E. Fault-zone properties and earthquake rupture dynamics Amsterdam: Elsevier, pp. 308 (2009).

[2] Rowe CD, Kirkpatrick JD, Brodsky EE (2012). Fault rock injections record paleo-earthquakes. Earth Planetary Science Letters.

[3] Bizhu H, Xiufu Q (2015). Advances and Overview of the Study on Paleoearthquake Events: A Review of Seismites. Acta Geologica Sinica (English Edition).

[4] Sibson RH (1975). Generation of pseudotachylyte by ancient seismic faulting. Geophysical Journal International.

[5] Rowe CD, Griffith WA (2015). Do faults preserve a record of seismic slip: A second opinion. Journal of Structural Geology.

[6] Kirkpatrick JD (2012). The depth of pseudotachylyte formation from detailed thermochronology and constraints on coseismic stress drop variability. Journal of Geophysical Research.

[7] Cox SF, Munroe SM (2016). Breccia formation by particle fluidization in fault zones: implications for transitory, rupture-controlled fluid flow regimes in hydrothermal systems. American Journal of Science.

[8] Smith SAF, Collettini C, Holdsworth RE (2008). Recognizing the seismic cycle along ancient faults: CO_2_-induced fluidization of breccias in the footwall of a sealing low angle normal fault. Journal of Structural Geology.

[9] Han R (2007). Seismic slip record in carbonate bearing fault zones: an insight from high-velocity friction experiments on siderite gouge. Geology.

[10] Hirose T, Bystricky M (2007). Extreme dynamic weakening of faults during dehydration by coseismic shear heating. Geophysical Research Letters.

[11] De Paola N (2011). Fault lubrication and earthquake propagation in thermally unstable rocks. Geology.

[12] Kameda J (2011). Smectite to chlorite conversion by frictional heating along a subduction thrust. Earth Planetary Science Letters.

[13] Kirkpatrick JD (2013). Silica gel formation during fault slip: evidence from the rock record. Geology.

[14] Suchy V, Frey M, Wolf M (1997). Vitrinite reflectance and shear-induced graphitization in orogenic belts: a case study from the Kandersteg area, Helvetic Alps, Switzerland. International Journal of Coal Geology.

[15] O’Hara K (2004). Paleo-stress estimates on ancient seismogenic faults based on frictional heating of coal. Geophysical Research Letters.

[16] Goldfinger C (2003). Holocene earthquake records from the Cascadia subduction zone and Northern San Andreas fault based on precise dating of offshore turbidites. Annual Review of Earth and Planetary Sciences.

[17] Pec M (2012). Origin of pseudotachylites in slow creep experiments. Earth Planetary Science Letters.

[18] Cowan DS (1999). Do faults preserve a record of seismic slip? A field geologist’s opinion. Journal of Structural Geology.

[19] Keulen N (2007). Grain size distributions of fault rocks: A comparison between experimentally and naturally deformed granitoids. Journal of Structural Geology.

[20] Boullier A-M (2004). Textural evidence for recent co-seismic circulation of fluids in the Nojima fault zone, Awaji island, Japan. Tectonophysics.

[21] Okamoto A, Tsuchiya N (2009). Velocity of vertical fluid ascent within vein-forming fractures. Geology.

[22] Monzawa N, Otsuki K (2003). Comminution and fluidization of granular fault materials: implications for fault slip behavior. Tectonophysics.

[23] Hofmann BA (2004). Topography-driven hydrothermal breccia mineralization of Pliocene age at Grimsel Pass, Aar massif, Central Swiss Alps. Schweizerische Mineralogische und Petrographische Mitteilungen.

[24] Belgrano T, Berger A, Herwegh M (2016). Inherited structural controls on fault geometry, architecture and hydrothermal activity: an example from Grimsel Pass, Switzerland. Swiss Journal of Geoscience.

[25] Wehrens P (2016). Deformation at the frictional-viscous transition: Evidence for cycles of fluid-assisted embrittlement and ductile deformation in the granitoid crust. Tectonophysics.

[26] Rolland Y, Cox SF, Corsini M (2009). Constraining deformation stages in brittle-ductile shear zones from combined field mapping and ^40^Ar/^39^Ar dating: The structural evolution of the Grimsel Pass area (Aar Massif, Swiss Alps). Journal of Structural Geology.

[27] Egli D (2018). Structural characteristics, bulk porosity and evolution of an exhumed long-lived hydrothermal system. Tectonophysics.

[28] Wehrens P (2017). How is strain localized in a mid-crustal basement section? Spatial distribution of deformation in the Aar massif (Switzerland). Journal of Structural Geology.

[29] Berger A (2017). Microstructures, mineral chemistry and geochronology of white micas along a retrograde evolution: An example from the Aar massif (Central Alps, Switzerland). Tectonophysics.

[30] Bergemann C (2017). Th-Pb ion probe dating of zoned hydrothermal monazite and its implications for repeated shear zone activity: An example from the Central Alps, Switzerland. Tectonics.

[31] Sonney R, Vuataz F-D (2008). Properties of geothermal fluids in Switzerland: A new interactive database. Geothermics.

[32] Waber N (2017). Constraints on Evolution and Residence Time of Geothermal Water in Granitic Rocks at Grimsel (Switzerland). Procedia earth and planetary science.

[33] Frenzel M, Woodcock NH (2014). Cockade breccia: Product of mineralisation along dilational faults. Journal of Structural Geology.

[34] Genna A (1996). Genesis of cockade breccias in the tectonic evolution of the Cirotan epithermal gold system, West Java. Canadian Journal of Earth Science.

[35] Niemeijer AR, Spiers CJ (2006). Velocity dependence of strength and healing behaviour in simulated phyllosilicate-bearing fault gouge. Tectonophysics.

[36] Nakatan M, Scholz CH (2004). Frictional healing of quartz gouge under hydrothermal conditions: 1. Experimental evidence for solution transfer healing mechanism. Journal of Geophysical Research.

[37] Micklethwaite S (2009). Mechanisms of faulting and permeability enhancement during epithermal mineralisation: Cracow goldfield, Australia. Journal of Structural Geology.

[38] Tenthorey E, Cox SF (2006). Cohesive strengthening of fault zones during the interseismic period: An experimental study. Journal of Geophysical Research.

[39] Uysal T (2011). Seismic cycles recorded in late Quaternary calcite veins: Geochronological, geochemical and microstructural evidence. Earth and Planetary Science Letters.

[40] Ergun S (1952). Fluid flow through packed columns. Chemical Engineering Progress.

[41] De Luca G, Di Carlo G, Tallini M (2018). A record of changes in the Gran Sasso groundwater before, during and after the 2016 Amatrice earthquake, central Italy. Scientific Reports.

[42] Chiaraluce L (2017). The 2016 Central Italy Seismic Sequence: A First Look at the Mainshocks, Aftershocks, and Source Models. Seismological Research Letters.

[43] Nuriel P (2012). U-Th dating of striated fault planes. Geology.

[44] Hansman RJ (2018). Absolute ages of multiple generations of brittle structures by U-Pb dating of calcite. Geology.

[45] Goodfellow BW (2017). Palaeocene faulting in SE Sweden from U Pb dating of slickenfibre calcite. Terra Nova.

[46] Ault AK (2015). Linking hematite (U-Th)/He dating with the microtextural record of seismicity in the Wasatch fault damage zone, Utah, USA. Geology.

[47] Diehl T (2018). Earthquakes in Switzerland and surrounding regions during 2015 and 2016. Swiss Journal of Geoscience.

[48] Schneider CA, Rasband WS, Eliceiri KW (2012). NIH Image to ImageJ: 25 years of image Analysis. Nat. Methods.

[49] Heilbronner, R. & Barrett, S. *Image Analysis in Earth Sciences: Microstructures and textures of earth Materials*. Springer (2014).

[50] Akinfiev NN, Diamond LW (2009). A simple predictive model of quartz solubility in water–salt–CO_2_ systems at temperatures up to 1000 °C and pressures up to 1000 MPa. Geochimica et Cosmochimica Acta.

