# Publisher Correction: Cockade structures as a paleo-earthquake proxy in upper crustal hydrothermal systems

**DOI:** 10.1038/s41598-019-53788-w

**Published:** 2019-11-25

**Authors:** Alfons Berger, Marco Herwegh

**Affiliations:** Institute of Geological Science University Bern, Baltzerstr. 1+3, 3012 Bern, Switzerland

Correction to: *Scientific Reports* 10.1038/s41598-019-45488-2, published online 25 June 2019

Supplementary Information files 1 and 2 were omitted from the original version of this Article. This has been corrected in the HTML version of the Article; the PDF version was correct at time of publication.

