# Supplementary material for: Cockade structures as a paleo-earthquake proxy in upper crustal hydrothermal systems
Source: Sci Rep. 2019 Jun 25;9:9209. doi: 10.1038/s41598-019-45488-2 (PMC6592875; doi:10.1038/s41598-019-45488-2)
Supplement: Supplementary file 1 — Supplementary information 1 [file 41598_2019_45488_MOESM1_ESM.pdf]

Supplementary information 1 (Seismic rupturing)

Cockade structures as a paleo-earthquake proxy in upper crustal hydrothermal systems

by

Alfons Berger[1,*] and Marco Herwegh[1]

1: Institute of Geological Science University Bern,
Baltzerstr. 1+3,
3012 Bern
Switzerland

*: corresponding author, email: alfons.berger@geo.unibe.ch

We use the geometry of the Grimsel Breccia Fault, consisting of major brittle fault strands connected by linkage zones[24], and the related displacement to estimate a potential seismic moment. Figure 1a represents a schematic block model of the strands of the vertical strike slip fault connected by a steep linkage zone. In the case of a seismic rupture the active part in the linkage zone is a mode I fracture, which opens and is oriented at high angle to the slip direction. The volume of the open fracture relates to the fracture's lateral extend between the two fault strands as well as to the displacement (s) of the strands of the strike slip fault. These values allow estimating the seismic moment (M_0) by:

$$M_o = \mu As \quad \text{(A1)}$$

Where μ is the shear strength and A is the fault plane area activated during the earthquake, which is defined by shear plane length (B) times its width (W). The seismic moment can be converted into the more frequently used earthquake magnitude (M_w) by [52]:

$$M_w = 2/3\log(M_o)-10.7 \quad \text{(A2)}$$

The rupture-induced total volume change of the linkage zone's open fracture is given by (Fig. 1):

$$V_{tot} = WLs \quad \text{(A3)}$$

where W is the width of the opened fracture and L its length (see Fig. 1 of main text for relations between W, B, L and s). It is this fracture, which after rupturing contains a mixture of the injecting fluid and the fragments plugged off from the wall rock (later cockade cores). The remaining open pore space, i.e. the effective pore volume (V_{eff}), in the fracture therefore is:

$$V_{eff} = V_{tot} * \phi \quad \text{(A4)}$$

where ϕ is the porosity (values between 0-1). Just before rupturing the initial pressure (P_i) of the fluid is nearly lithostatic:

$$P_i = \rho gz \quad \text{(A5)}$$

with ρ as rock density, $g = 9.81\ ms^{-2}$ and z the depth of the rupture in the Earth's crust. With rupturing, volumetric strain increases owing to dilatancy, ϕ therefore increases and consequently the fluid pressure drops. The ratio between the new fluid pressure (P_f) and the initial pressure P_i is:

$$P_f/P_i = 1/(1+s) \quad \text{(A6)}$$

Both, the volume change (ΔV)

$$\Delta V = V_{tot} - V_{eff} \quad \text{(A7)}$$

and the pressure drop (ΔP)

$$\Delta P = P_i - P_f \quad \text{(A8)}$$

drive fluidization of the rock fragments in the open fracture. The velocity of the fluid and associated transport of the fragments/cockades in the fluid is controlled by these two parameters[7] (see also Appendix 3).

Table A1: compilation of used symbols

symbol	unit	definition	remarks
M_o	J	seismic Moment	
μ	Pa	shear strength	
A	m^2	area of the activated fault (=W*B)	
W	m	fracture width	Fig. 1 and A4
B	m	shear plane length	Fig. 1
L	m	fracture length	Fig. A4
s	m	displacement	Fig. 1
V_{tot}	m^3	total volume of the linkage zone fracture	
V_{eff}	m^3	effective volume of this fracture zone	
ϕ	-	porosity	fraction
P	Pa	pressure	
P_i	Pa	initial pressure	
P_f	Pa	new fluid pressure	
z	m	depth of the rupture	
c_{Si}	mol/ kg water	initial solubility (starting conditions)	
c_{Sf}	mol/ kg water	final solubility (crystallisation conditions)	
m	kg	mass	
V_{eff}^{water}	m^3	effective water volume	Fig. A4
V_{tube}	m^3	systems volume of the tube	Fig. A4
$V_{cockade}$	m^3	volume of the cockade	Fig. A4
l	m	length of the basal plane	Fig. A4
h	m	height	Fig. A4
η	Pas	fluid viscosity	
u_s	ms^{-1}	superfical velocity	
u_m	ms^{-1}	velocity	
ϑ		particle spericity	
d	m	particle diameter	
ρ_f	kgm^{-3}	density of the fluid	
ρ_p	kgm^{-3}	density of the particle	
u_c	ms^{-1}	sink velocity of immobile cement particles	
T	m	thickness of geopetal cements	
t_{mc}	s	time of fast flow rate to growth one cockade rim	given in min
t_{cs}	s	time of sedimentation of geopetal cements	given in a
