# Supplementary material for: Cockade structures as a paleo-earthquake proxy in upper crustal hydrothermal systems
Source: Sci Rep. 2019 Jun 25;9:9209. doi: 10.1038/s41598-019-45488-2 (PMC6592875; doi:10.1038/s41598-019-45488-2)
Supplement: Supplementary file 2 — Supplementary information 2 [file 41598_2019_45488_MOESM2_ESM.pdf]

Supplementary information 2 (Geometrical measurements)

Cockade structures as a paleo-earthquake proxy in upper crustal hydrothermal systems

by

Alfons Berger[1,*] and Marco Herwegh[1]

1: Institute of Geological Science University Bern,
Baltzerstr. 1+3,
3012 Bern
Switzerland

*: corresponding author, email: alfons.berger@geo.unibe.ch

We analyze two samples, representing a large and a small cockade layer with respective different cockade sizes (Fig. A1). Sample GR22 derived from the "Sidelhorn linkage zone" (see [24] for location) and has a thickness of the cockade layer of 10-20 cm. Sample GDP1-2 derived from Totensee area and has a cockade layer thickness of 5 mm. Quantitative image analysis on these samples delivered values for the size and growth rims of the cockades in these layers (see methods section). The particle sizes result in median values of 2.56 mm and 0.22 mm, respectively, for Gr22 and GDP1-2. Figure A1a, b shows the frequency size distribution of the measured cockade particles. The median value is indicated.
In addition, the thicknesses of each layer in the growth rims are estimated in several cockades in one sample (GR22; Fig. A1c). The average size of each growth layer is ~0.02 mm in this sample (Table A2). Selecting the cockade of one seismic cycle, the number of growth layers is the same (Fig. A1c). As discussed in the main text, the volumetric difference of the pure cockade core and the final cockade particle gives the bulk volumetric gain which is used for precipitation of the mobile cements representing a gain of ~35±11% (Fig. A1d).

Table A2: Measured geometries of the cockades

	unit	*GR22*	*GDP1-2*
median cockade size	mm	2.5	0.02
cockade rim thickness	mm	0.02	
geopetal thickness	mm	5.6	
final porosity	-	0.35	

The immobile cements are best documented in the geopetal textures (main text, Fig. 2). They represent filled sediment traps occurring in former high porous domains throughout the sample. In order to quantify the layer thicknesses of the fines settled out of the suspension, we measured the length perpendicular to the layering structures for the different layers (Fig. A1e). This results in an average layer thickness of 5.9 mm.

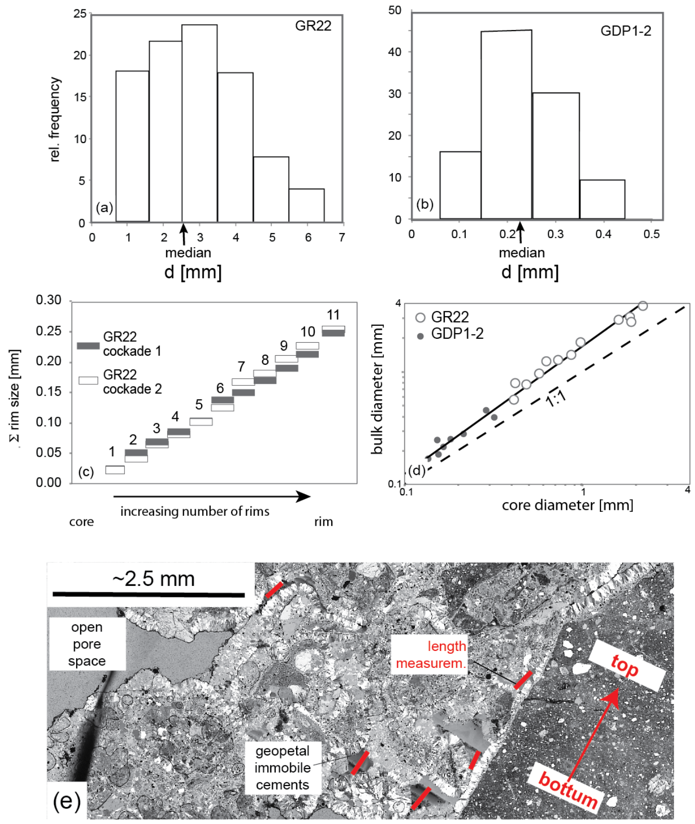

Figure A1: Measurement data of two key examples from the Grimsel breccia zone. (a) Cockade sizes of sample GR22, (b) cockade sizes of sample GDP1-2, (c) cumulative size curve for growth layers in two different cockade growth rims in sample GR22, (d) measurements of diameters of

cockade cores and entire cockades (core and rim) in samples GR22 and GDP1-2. The difference to the 1:1 line gives the overall size increase of the cockades owing to precipitation of mobile cements in the growth rims. (e) Illustrating the measurements of the layer thicknesses of the immobile geopetal cements as 1D length. The figure presents only a small part of the thin section.

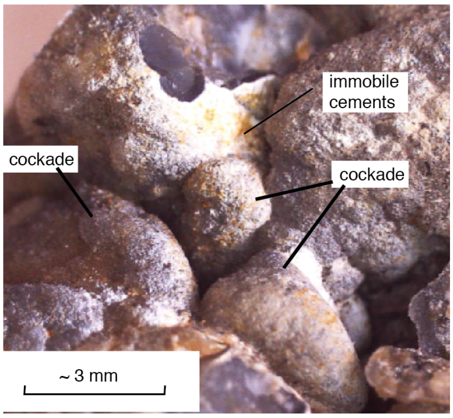

Figure A2: Photograph of several individual cockades with local preserved immobile cements (whitish). Note the 3D spherical shape of each cockade.
