# Supplementary material for: Cockade structures as a paleo-earthquake proxy in upper crustal hydrothermal systems
Source: Sci Rep. 2019 Jun 25;9:9209. doi: 10.1038/s41598-019-45488-2 (PMC6592875; doi:10.1038/s41598-019-45488-2)
Supplement: Supplementary file 3 — Supplementary information 3 [file 41598_2019_45488_MOESM3_ESM.pdf]

Supplementary information 3 (System dimensions)

Cockade structures as a paleo-earthquake proxy in upper crustal hydrothermal systems

by

Alfons Berger[1,*] and Marco Herwegh[1]

1: Institute of Geological Science University Bern,
Baltzerstr. 1+3,
3012 Bern
Switzerland

*: corresponding author, email: alfons.berger@geo.unibe.ch

Appendix 2 presents the geometrical aspects of different cockade layers. In addition, the pressure drop (ΔP) can be calculated by the difference between the lithostatic and hydrostatic pressures at a given earthquake depth (Appendix 1). This pressure drop is crucial with respect to the fluid velocity in the jet directly after rupturing as well as the associated solubility change being responsible for quartz precipitation in the mobile cockade cements (see main text body). In order to estimate fluid velocity as well as the growth rates of the mobile cockade cements, we calculate an example for an initial pressure (P_i) at a depth (z) of 6 km (~160 MPa and temperature of 150°C; initial geothermal gradient of 25°/km). Just before the earthquake, we assume the fluid to occur in isolated pores and therefore to be close to lithospheric pressure. Owing to the fracturing and volume increase during the earthquake the fluid pressure drops imposing a flow of the fluid (see main text). Pressure drop and flow last until being equilibrated with the ambient pressure condition, which at minimum is the hydrostatic pressure at corresponding depth in the crust. However, the real value is higher, due to the large fluid-pressure drop during the earthquake. At the limit, the fluid pressure after rupturing can be close to zero due the newly formed fracture space. In this case, the pressure drop would be as large as the lithostatic pressure[51]. This change in fluid pressure is isoenthalpic, because of the short time interval of the earthquake. For a pure aqueous, isoenthalpic fluid, the solubility changes from the state at P_i and P_f reducing the initial solubility (c_{si}) to a new solubility (c_{sf}). The bulk difference in solubility (Δc_S) therefore is:

$$\Delta c_S = c_{si} - c_{sf} \qquad \text{(A9)}$$

It is this bulk difference in solubility, which promotes quartz precipitation of the mobile cockade cements. The equation of state of aqueous fluids[50] using the software "LONER" (University of Leoben; R. Bakker) allows for the calculation of Δc_S. Additionally, the effect of the temperature drop because of the isoenthalpic behavior during the fast pressure drop (Joule Thomson effect) needs to be considered. The Joule Thomsen factors are taken from http://webbook.nist.gov/. Figure A3 shows the P-T space and associated solubility changes for given pressure drops.

For the following considerations, we reduce in a first step the system to one representative cockade and the required fluid volume for only this specific cockade. In order to precipitate the amount of SiO_2 measured ($m_{cockade}$) in one growth layer of a cockade rim, the effective amount of water (m_{eff}) and dissolved SiO_2 ($m_{\Delta cS}$) necessary at given Δc_S can be derived by:

$$m_{eff}^{water} = \frac{m_{\Delta cs}^{water} * m_{cockade}^{SiO2}}{m_{\Delta cs}^{SiO2}} \qquad \text{(A10)}$$

This can be transferred into volumes by using densities of quartz (2650 kgm^{-3}) and aqueous fluid (1000 kgm^{-3}), yielding in the effective water volume (V_{eff}) required for the rim precipitation.

$$V_{eff}^{water} = \frac{V_{\Delta cs}^{water} * V_{cockade}^{SiO2}}{V_{\Delta cs}^{SiO2}} \qquad \text{(A11)}$$

To constrain the hypothetical dimensions of the system (fluid plus cockade of the one cockade model system), we consider a vertical water column, defining the system's volume (V_{tube}). V_{tube} is defined by:

$$V_{tube} = V_{cockade} + V_{eff} \qquad \text{(A12)}$$

where $V_{cockade}$ is the volume of the entire cockade (core and growth layer). The dimensions of V_{tube} can be expressed by a basal plane (l^2) times a height (h). To constrain the basal plane, we assume that the lengths have to be larger than the diameter of the cockade to guarantee a free cockade movement in the water column and therefore calculate a unit volume (V_{unit}) for a single cockade including the assumed porosity (ϕ)/pore volume (V_ϕ):

$$V_{unit} = \frac{V_{cockade}}{(1-\phi)} = V_{cockade} + V_\phi \qquad \text{(A13)}$$

Under the simplified assumption of a cube-like shape, whose base and top plane correspond to the base of the vertical water column, the length (l) of the base line is:

$$l = \sqrt[3]{V_{unit}} \quad \text{(A14)}$$

Hence h can be derived by:

$$h = \frac{V_{eff}^{water}}{l^2} \quad \text{(A15)}$$

In contrast to the initial model assumption of a single cockade system, the cockade microstructures indicate that a finite number of cockades were in suspension. The volumes of these cockades therefore increase the estimated V_{tube} to $V_{tube'}$ by

$$V_{tube'} = V_{tube} * (1 - \phi) \quad \text{(A16)}$$

The new height (h') of the vertical tube therefore is:

$$h' = \frac{V_{tube'}}{l^2} \quad \text{(A17)}$$

Filling up the complete system with particles, the amount of water has to multiply by the number of cockade-cores available in the representative space. This can be approximated by:

$$V_{tube"} = V_{tube'} * \text{number of cockades} \quad \text{(A18)}$$

The necessary number of cockade is given by the selected porosity and the size of the cockades. Therefore, the new height (h'') of the vertical tube is:

$$h" = \frac{V_{tube"}}{l^2} \quad \text{(A19)}$$

Taking again our representative cockade, V_{eff}^{water} has to pass this reference cockade during upflow in order to guarantee the precipitation of the required amount of quartz (see above). This means, the V_{tube} and $V_{tube'}$ have to be added on top of the reference cockade for, respectively, the single and multiple cockade case. This requires new space, which we consider to be the upper part of the fracture. Therefore the minimum length (single W_{min} or multiple cockades W'_{min}) of the entire fracture can be estimated by:

$$W_{min} = 2*h \text{ or } W'_{min} = 2*h' \text{ or } W''_{min} = 2*h'' \quad \text{(A20)}$$

These values can now be used to calculate the magnitude of the rupture following equations A1-A3, where the displacement is taken from the size of the cockade layer and the shear plane length (B) have to assumed from the field. An example for sample GR22 is given in Figure A5.

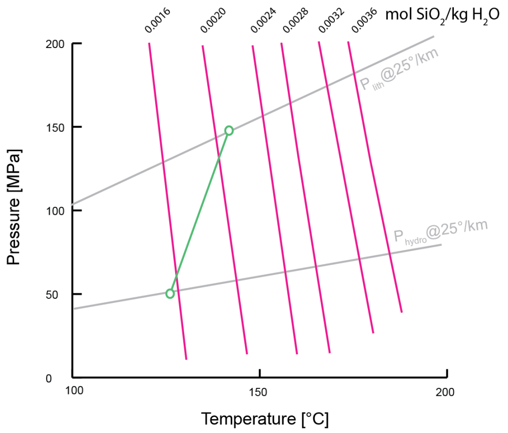

Fig. A3: Diagram showing the calculated SiO_2 solubility: Red lines: contoured solubility of SiO_2 in pure water (contoured with data calculated by software "LONER"). Grey lines hydrostatic and

lithostatic pressures, respectively. Green line: example of a solubility change as a consequence of one pressure drop including isoenthalpic cooling.

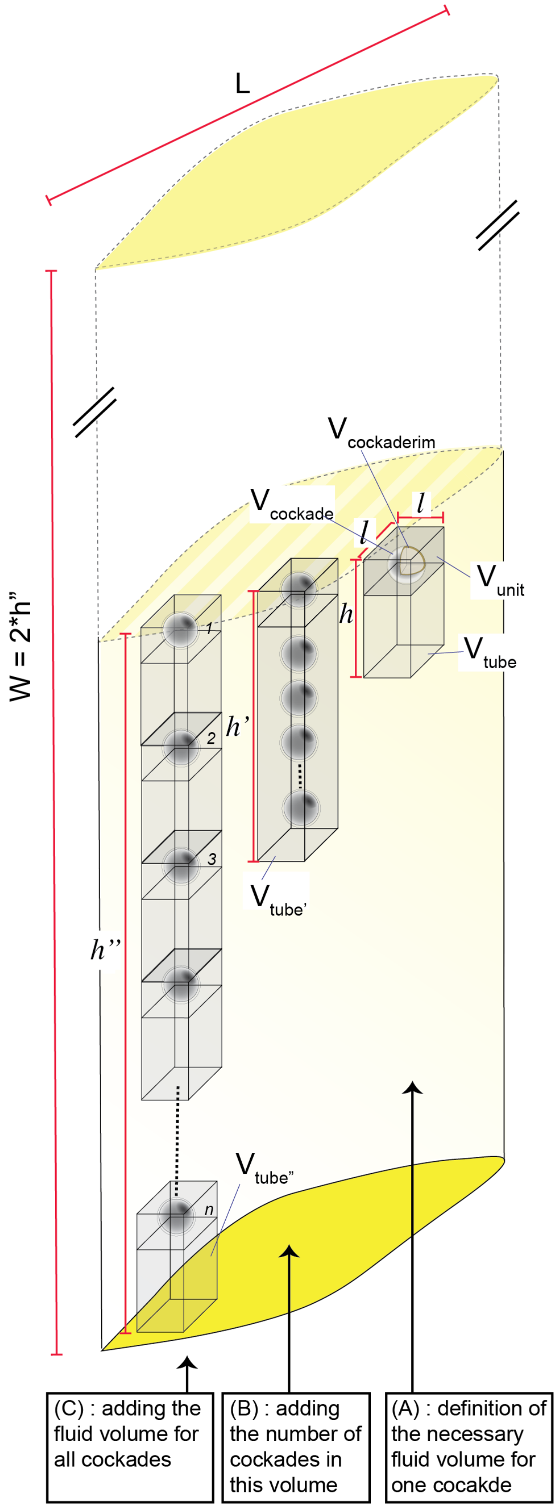

Fig. A4: Schematic view on the model and the used dimensions. The column (A) shows the calculated fluid volume necessary to growth one cockade rim. The amounts of cockades are added (shown in column (B)), which are calculated from their size and the assumed porosity. All cockades need the amount of fluid as shown in (A), which are added for the amount of cockades in this volume (indicated in column (C)). This result in a representative volume, which is defined by the size “l” and the height “ h” ”. This value h” is used to calculated the minimum width of the fault (W).

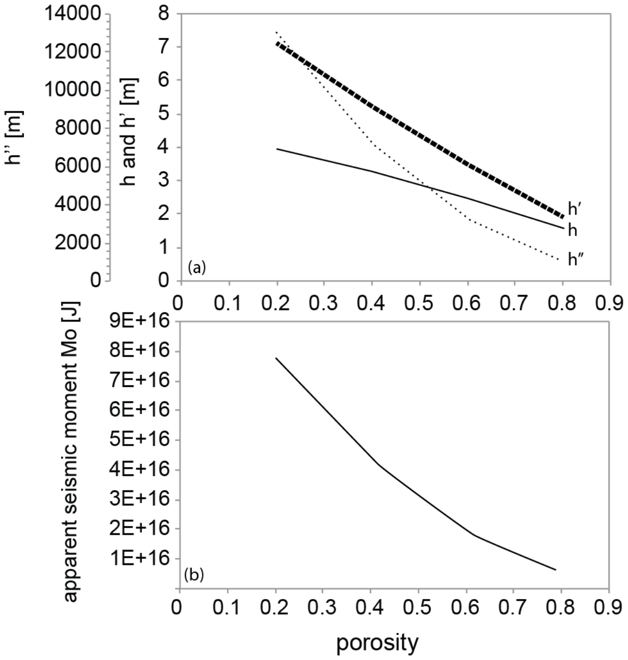

*Fig. A5: The variations of h and seismic moment with the porosities in a given cockade layer. (a) Calculated h, h', h" in relation to measured or inferred porosity. (b) Calculated related seismic moments (s: 0.1m; B: 1000m, W =h"*2 (see values of h" in (a)), μ: 30GPa).*
