# Supplementary material for: Cockade structures as a paleo-earthquake proxy in upper crustal hydrothermal systems
Source: Sci Rep. 2019 Jun 25;9:9209. doi: 10.1038/s41598-019-45488-2 (PMC6592875; doi:10.1038/s41598-019-45488-2)
Supplement: Supplementary file 4 — Supplementary information 4 [file 41598_2019_45488_MOESM4_ESM.pdf]

Supplementary information 4 (Time constraints)

Cockade structures as a paleo-earthquake proxy in upper crustal hydrothermal systems

by

Alfons Berger[1,*] and Marco Herwegh[1]

1: Institute of Geological Science University Bern,
Baltzerstr. 1+3,
3012 Bern
Switzerland

*: corresponding author, email: alfons.berger@geo.unibe.ch

We defined the dimensions of the fracture allowing now investigating associated rates. In this sense, three major stages of the seismic cycle need to be distinguished: (i) main rupture, (ii) aftershocks and (iii) interseismic period (Fig. 3). Associated cement types and precipitation stages strongly depend on the fluid velocity. This relation be expressed by the Ergun-equation[7]:

$$\frac{\Delta P}{W} = \frac{150\eta(1-\phi)^2 u_s}{\vartheta^2 \phi^3 d^2} + \frac{1.75(1-\phi)\rho u_s^2}{\vartheta \phi^3 d} \quad \text{(A21)}$$

ΔP is the fluid pressure drop, W is the height of the layer, η is the fluid viscosity, ϑ is the particle sphericity, ϕ is the porosity, d is the particle diameter, ρ_f is the density of the fluid, u_s is the superficial velocity of the fluid. The superficial velocity is defined as the ratio of the volumetric flow rate of the fluid to the cross sectional area of the layer. The equation shows the dependency of the pressure drop, porosity and particle size to the flow velocity.
The relationship can be simplified by assuming pure earth acceleration, which results in:

$$g(\rho_P - \rho_f) = \frac{150\eta(1-\phi)^2 u_m}{\phi^3 d^2} + \frac{1.75\rho u_m^2}{\phi^3 d} \quad \text{(A22)}$$

The resulting velocity (u_m) defines the minimum conditions required to bring cockades with a given size in suspension. Larger particles will not be mobilized by the injecting fluid.
The time (t_{mc}) of mobile cockade rim cementation can then be derived by knowing the minimum fracture length (eq. A20) and the flow velocity (u_m):

$$t_{mc} = \frac{W_{min}}{u_m} \quad \text{(A23)}$$

The resulting fast times give a first indication on the short time intervals during the earthquake. In contrast to the mobile cockade cements, the geopetal cements indicate time intervals of zero or very low fluid velocity during which the fines in suspension settle. Hence the represent stationary fluid stages. The geopetal cements therefore document interseismic periods being devoid of any pressure drops, i.e. fluid flow. The sedimentation rate of the fines can be calculated from Stoke's law:

$$u_c = \frac{2}{9} * \frac{(\frac{d}{2})^2 * g * (\rho_p - \rho_f)}{\eta} \quad \text{(A24)}$$

The sedimentation time (t_{cs}) depends on the above described sink rate (u_c), the size of the particles (d) and the thickness of the layer (T) and the length of the water column, which is given by the minimum length of the fracture (W). The ultrafine particles in suspension are homogenously distributed. The settling distance in the water column has therefore to be derived by integrating between the maximum and minimum length scale of the fracture, which results in an average length of $W_{min}/2$.

$$t_{cs} = \frac{\mathrm{T}}{\mathrm{d}} * \frac{\frac{W_{min}}{2}}{u_c} \quad \text{(A25)}$$

With the measured thickness (see Fig. A1, Table A2), the measured particle size inside the geopetal cements and the calculated length, we can estimate the sedimentation time (t_{cs}) necessary to form the geopetal cements.

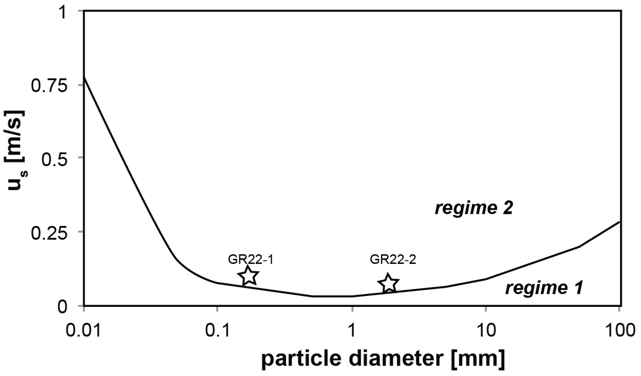

Fig. A6: Boarder between a minimum fluidization velocity; and their overstepping. In regime 1 (below minimum fluidization), particles will remain as an aggregate, whereas by the overstepping of velocity (um) particles start to move in suspension (see [7] and eq. A21). In order to calculate a minimum velocity of the fluid using porosity (φ) of 0.6; particle density of 2650 kgm^{-3}; fluid density of 1000 kgm^{-3} and viscosity (η) of 0.00012Pas we result a minimum velocity of 0.1ms^{-1}.

Table A3: Parameters and calculated results to exemplary illustrate the mass fluxes and required time intervals

		unit	*core*	*cement rim*		
diameter/thickness	d	mm	2.5	0.02		
volume	$V_{cockade}$	mm^3	8.18	0.399		
mass		mol qtz		1.75E-5		
example for porosities				***0.4***	***0.6***	***0.8***
solubility change ΔP: 110-50MPa	Δc_S	mol/kg H_2O		0.000945		
effective water volume necessary	V	mm^3		18’624	18’624	18’624
representative volume	V_{unit}	mm^3		13.64	18.62	40.91
minimum fracture length	h	m		3.26	2.49	1.57
	$V_{tube'}$	mm^3		29’798	26’074	22’349
minimum fracture length at ϕ	h’	m		5.22	3.49	1.88
minimum fluid flow velocity	u_m	ms^{-1}		~0.1	~0.1	~0.1
time require to pass by this fluid	t_{mc}	min		1.2	0.6	0.3
	h’’	m		7131	3174	857
time for immobile cements	t_{cs}	a		22’243	9’943	2’684
